# The Management of Coronary Artery Disease in Ethiopia: Emphasis on Revascularization

**DOI:** 10.4314/ejhs.v31i2.27

**Published:** 2021-03

**Authors:** Bekele Alemayehu Shashu

**Affiliations:** 1 MD, Internist, Interventional Cardiologist, Associate Professor of Medicine, Addis Ababa University

**Keywords:** coronary artery disease, revascularization

## Abstract

Cardiovascular diseases are number one cause of death worldwide. Over half of the cardiovascular diseases, 51%, are due to coronary artery disease. Coronary artery disease is a pathological process characterized by atherosclerotic plaque accumulation in the epicardial coronary arteries. Rupture of the fibrous cap of the plaque causes the majority of the deaths due to myocardial infarction. Angina pectoris is a discomfort in the chest or adjacent areas caused by myocardial ischemia usually precipitated by exertion. In acute coronary syndrome, the chest discomfort is either of low threshold or appears at rest and when it evolves on the background of established angina pectoris, the discomfort becomes more frequent and prolonged. Exercise electrocardiography which has been the most frequently used non-invasive test to diagnose obstructive coronary artery disease is currently shown to have inferior diagnostic performance compared with diagnostic imaging tests. The pivotal tests in patients presenting with clinical features of acute coronary syndrome are electrocardiography and determination of serum troponin I and/or T. Revascularization is the mainstay of treatment in patients with acute coronary syndrome. In chronic coronary syndrome, on top of optimal medical treatment, revascularization reduces mortality in:- 1) left main stenosis, 2) three-vessel coronary artery disease, particularly with ejection fraction of less than 40%, 3) two vessel disease with more than 75% stenosis of the proximal left anterior descending coronary artery disease.

## Introduction

Cardiovascular diseases (CVD) are number one cause of death worldwide (1). An estimated 17.9 million people died from CVDs in 2016, representing 31% of all global deaths. Of these deaths, 85% are due to myocardial infarction (MI) and stroke. Over three quarters of CVD deaths take place in low- and middle-income countries. Out of the 17 million premature deaths (under the age of 70) due to non-low- and middle-income countries, of which 37% were caused communicable diseases (NCD) in 2015, 82% were in by CVDs (2). Global deaths from CVD increased by 41% between 1990 and 2013 despite a 39% decrease in age-specific death rates; this increase was driven by a 55% increase in mortality due to the aging of populations and a 25% increase due to population growth. The relative contributions of these drivers varied by region; only in Central Europe and Western Europe did the annual number of deaths from CVD actually declined (1). From 2011 to 2025, the projected cumulative economic loss from all NCD is $7.28 trillion in low and middle-income countries (LIMC), CVD accounts for nearly 50% of this projected loss. It is also projected that reducing CVD mortality by 10% in LMIC would result in a $377 billion reduction in economic loss from 2011 to 2025 (3).

Over half of the CVDs, 51%, are due to coronary heart disease (CHD), the remaining is caused by stroke, heart failure, hypertension, arterial diseases and others (4). Coronary artery disease (CAD) is a pathological process characterized by atherosclerotic plaque accumulation in the epicardial arteries. The disease can have long, stable periods but can also become unstable at any time, typically due to an acute atherothrombotic event caused by plaque rupture or erosion, rarely due to erosion through calcified nodule or intraplaque hemorrhage (5). Although the disease is chronic, it is most often progressive, and hence serious, even in clinically apparently silent periods. The dynamic nature of the CAD process results in various clinical presentations, which can be conveniently categorized as either acute coronary syndromes (ACS) or chronic coronary syndromes (CCS).

In the Ethiopian context, the most frequently encountered clinical scenarios in patients with suspected or established CAD are: (i) patients with heart failure with unrecognized or misdiagnosed preceding acute MI (ii) stable anginal symptoms, and/or angina equivalents like dyspnea, faintness, fatigue and others. (iii) acute coronary syndrome (iv) patients presenting with malignant arrhythmia and/or sudden death. According to our study and daily practice, the former is regrettably most frequent (6). Regardless, no countrywide studies have been done on CVD, except for some hospital based studies and a few community surveys showing CVD as an important cause of morbidity and mortality. In studies done in the late 1960s and 1970s, CVD accounted for 4–13% of the medical admissions to hospitals in Addis Ababa (7). From September 1975 to August 1979, a total of 5667 patients were admitted to the medical wards of the Tikur Anbessa Specialized Hospital (TASH), the largest teaching hospital in the country, where 381 (6.7%) admissions were due to CVD (8). Recent studies indicated enormous increase in the number of CVD cases. Clinical analysis done in the 1990s documented acute MI alone as a cause for 8.8 % of admissions to the medical intensive care unit of the same hospital making it the third commonest cause of admission after severe malaria and diabetic ketoacidosis (9). We also reported that among patients who have undergone coronary angiography on the bases of suspected CAD at Addis Cardiac Hospital, 76% had evidences of the disease, mostly multi-vessel involvement (6).

**Pathophysiology**

**Figure 1:**
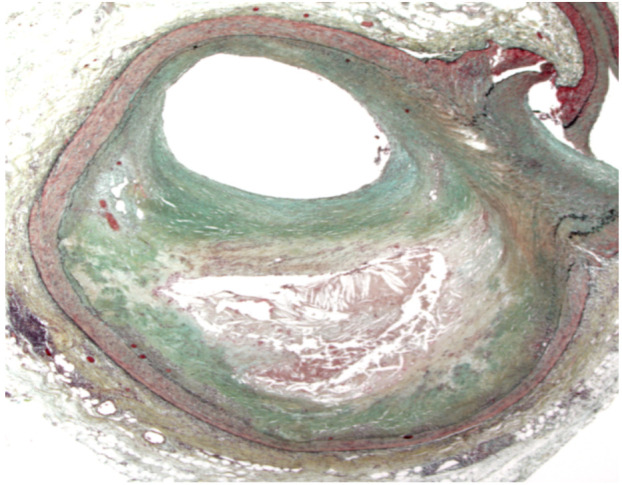
A Cross-sectional view of an atherosclerotic plaque

An atherosclerotic plaque is composed of a fibrous cup, central lipid core, cellular elements including (lipid laden macrophages, T lymphocyte and smooth muscle cells), some degree of calcification and thrombotic material (Fig 1). A fibrous cap typically overlies the lipid-rich center also known as the necrotic core of an atheromatous plaque. Findings from pathologic studies have revealed that plaques presenting acutely are not occlusive enough to elicit symptoms before the acute event. The plaque at the sites of the culprit lesion of an acute MI with coincidental preceding coronary angiogram often does not show stenosis sufficiently severe to limit flow (10). In a prospective angiographic study involving patients undergoing percutaneous coronary intervention (PCI) for CCS, only half the subsequent events arose from lesions with sufficient stenosis to have warranted intervention (11). This dissociation between the degree of stenosis and the propensity to provoke ACS helps to explain why MI often occurs without being signaled by the demand-induced symptoms of angina that would result from a high-grade stenosis. Technologies that permit cross-sectional imaging of the coronary arteries, such as intravascular ultrasonography or CT angiography, underscore the pathological observation that the outward expansion of atherosclerotic arteries accommodates the growth of plaque for much of its life history (10). Compensatory enlargement (outward expansion) of the artery during plaque growth can conceal a considerable burden of atheroma by preventing stenosis and thereby obscuring signs and symptoms of ischemia. Sizable plaques can reside in the walls of affected arteries without detection on arteriograms and no any warning symptoms to the patient. Luminal stenosis occurs relatively late in the process of atherogenesis, when plaque growth outstrips the ability of the artery to compensate by expanding outward (12).

Rupture of the fibrous cap of the plaque causes the majority of the deaths due MI; superficial erosion of a coronary artery accounts for most of the balance of fatal events. Autopsy studies have shown that erosion through the intima of a calcified nodule and intraplaque hemorrhage each trigger only a small percentage of ACS (13).

**Figure 2:**
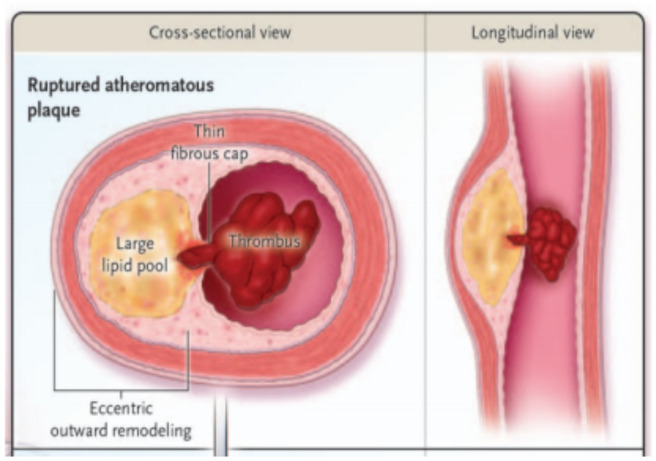
Cross-sectional and longitudinal view of a raptured plaque

Investigations on pathophysiologic mechanisms have focused on plaque rupture as the most common cause of fatal and/or hemodynamically grave ACS (Fig 2). The fibrous cap stands between the blood compartment, with its latent coagulation factors, and the lipid core. Morphometric studies have identified the characteristics of unstable plaques that have ruptured and caused fatal MI which often have thin fibrous caps (14). Ruptured plaques also tend to have large lipid cores, abundant inflammatory cells, few smooth muscle cell as well as punctate calcification (15).

**Figure 3:**
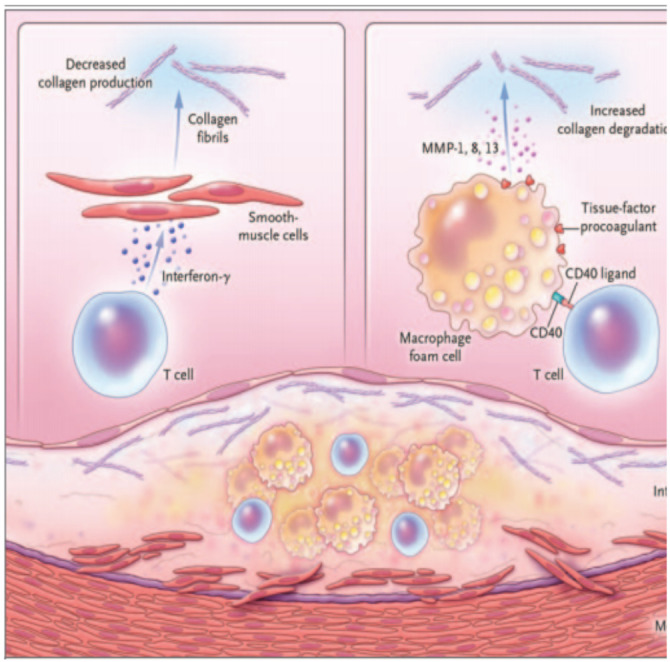
Inflammatory Pathways Predisposing Coronary Arteries to Rupture and Thrombosis

The fibrous cup protects the plaque from rupture through its tensile strength obtained from interstitial collagen synthesized by smooth-muscle cells. The association between thinning of the fibrous cap and fatal plaque rupture ignited in vivo studies focusing on collagen biosynthesis. A study of the control of collagen biosynthesis by human vascular smooth-muscle cells in culture revealed that exposure to interferon-γ produced by activated T cells strongly inhibited the ability of smooth-muscle cells to make the new collagen required to repair and maintain the integrity of the fibrous cap (16). Another study showed an inverse correlation between T-cell accumulation in human atherosclerotic plaques and the messenger RNA that encodes the precursor of interstitial collagen, an observation that supports the relevance in vivo of the profound inhibition of new collagen synthesis by a T-cell-derived mediator (17). Rate of collagen degradation is also important factor in the thinning of the fibrous cup. A family of enzymes, matrix-metalloproteinase (MMP), is over produced in the plaque proper. Studies of the regulation of MMP production by human macrophages have shown that the T-cell-derived cytokine CD40 ligand (CD154) boosts the production of interstitial MMP by human macrophages (18). Thus, cross-talk between T cells and macrophages inhibits the synthesis and augments the degradation of interstitial collagen (Fig 3).

Superficial erosion of coronary atheromata causes approximately 20 to 25% of cases of fatal acute MI (14). Erosions differ from rupture lesions, as there is denudation of endothelial cells without fibrous cap disruption i.e. there is no communication between necrotic core and the lumen. Many lesions that cause coronary thrombosis because of superficial erosion lack prominent inflammatory infiltrates; such plaques exhibit proteoglycan accumulation. The primary cellular characteristics of plaque erosion include an abundance of smooth muscle cells and proteoglycan matrix, and absence of surface endothelium without a prominent lipid core and few macrophages and T-lymphocytes close to the lumen (19). The underlying lesion morphology also differs from rupture since it involves early lesions such as pathologic intimal thickening or fibroatheromas without an extensive necrotic core, hemorrhage, or calcification. The mechanisms of superficial erosion have received much less attention than those involved in the rupture of the fibrous cap. Programmed cell death (i.e. apoptosis) of endothelial cells could contribute to their desquamation; oxidative stress can also promote endothelial apoptosis ((20).

**Clinical manifestation**

Angina pectoris is a discomfort in the chest or adjacent areas caused by myocardial ischemia. It is usually precipitated by exertion but may also be initiated by emotional distress. It is frequently described as constricting, suffocating, crushing, heavy, and squeezing type. In other patients the quality of the sensation can be vague and described as a mild pressure-like discomfort, tightness, an uncomfortable numbness, or a burning sensation. The site of the discomfort is usually retrosternal, but radiation is common and generally occurs down the ulnar surface of the left arm; the right arm and the outer surfaces of both arms may also be involved. Epigastric discomfort alone or in association with chest pressure may occur and can be disregarded as dyspepsia. Anginal discomfort above the mandible or below the epigastrium is rare. Anginal equivalents (i.e. symptoms of myocardial ischemia other than angina), such as dyspnea, faintness, fatigue, and frequent belching, are more commonly seen in women, older adults, patients with diabetes and with chronic kidney disease.

In ACS patients, the chest discomfort is either of low threshold or appears at rest and when it evolves on the background of established stable angina pectoris, the discomfort becomes more frequent and prolonged. Thoughtful characterization of the presenting complaint is the most important aspect of the diagnostic workup of a patient suspected to have ACS. Acute chest pain is one of the most common reasons for seeking care in the emergency department, accounting for approximately 10% of all visits. After diagnostic evaluation, only 10% to 15% of patients with acute chest pain actually have ACS (22). The difficulty lies in identifying these patients out of the large group with similar presentations. Discriminating ACS from those with other life-threatening conditions like aortic dissection, cardiac tamponade, pulmonary embolism and others is also critical. Therapeutic interventions for acute MI could be catastrophic if one of the alternative life-threatening conditions is misdiagnosed.

**Diagnostic workup**

Patients suspected to have CCS should undergo tests targeting risk factors of CVD as well as conditions that are important in triggering symptoms. Full blood count with hemoglobin, fasting blood glucose plus hemoglobin A1c (HbA1c), creatinine and lipid profile including low density lipoprotein cholesterol (LDL) measurements are essential. The likelihood of obstructive CAD is influenced by the prevalence of the disease in the population and the clinical presentation of the individual patient. A simple predictive model designed by Diamond and Forrester, in 1979, has been in use to estimate the pre-test probability (PTP) of obstructive CAD based on age, sex and nature of symptoms (22). Exercise ECG is used to diagnose obstructive CAD in those with intermediate pre-test probability (15 -85 %) applying this model as updated by Genders and his group in 2011, endorsed by European Society of Cardiology (Table 1) (23).

**Table 1:** Clinical pre-test probabilities in patients with stable chest pain symptoms

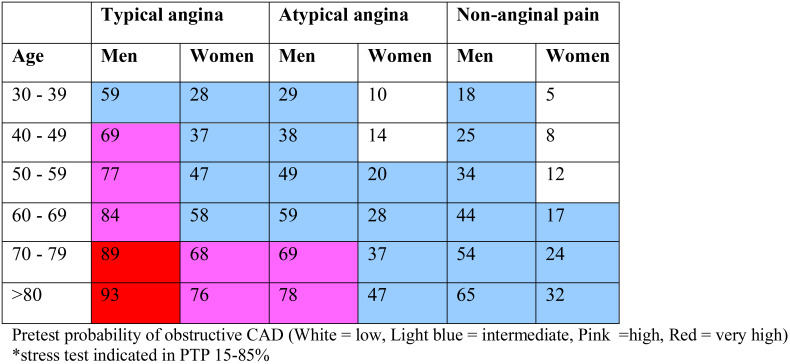

Pretest probability of obstructive CAD (White = low, Light blue = intermediate, Pink =high, Red = very high)

*stress test indicated in PTP 15–85%

However, overestimation of PTP by the Diamond-Forrester model is an important contributory factor to low diagnostic yield of non-invasive and invasive testing (24). The most frequently used non-invasive test, exercise ECG, has also inferior diagnostic performance due to limited power to rule-in or rule-out obstructive CAD compared with diagnostic imaging tests: the anatomic imaging, coronary CT angiography (CCTA) or functional imaging including myocardial perfusion studies, cardiac MRI (CMR) or stress echocardiography (25). Current guidelines recommend the use of an imaging diagnostic test instead of exercise ECG as the initial test to diagnose obstructive CAD (26); however, exercise ECG may be considered as an alternative if any of the imaging tests are not available. It also provides useful information regarding prognosis through monitoring for the strength of symptoms, extent of ST-segment changes, exercise tolerance, arrhythmia, and blood pressure response throughout the procedure and recovery period.

The new set of PTPs designed by the European Society of Cardiology (ESC) (table 2) is believed to substantially reduce the need for non-invasive and invasive tests in patients with suspected CAD. Studies have shown that outcomes in patients classified with the new PTP to be <15% is good (annual risk of cardiovascular death or MI < 1%) (25). It is safe to defer routine testing in patients with PTP <15% (28), thus reducing unnecessary procedures and costs while patients with PTP 15% or higher are candidates for imaging diagnostic tests. Because it can detect subclinical atherosclerosis, CCTA is preferred for patients with low PTP to rule-out obstructive CAD whereas functional imagines are chosen when the PTP is high to rule-in obstructive CAD (26). Considering the prevailing circumstances, stress echocardiography and CCTA are applicable in Ethiopia with relatively lower costs for the establishment of facility and training of man power.

**Table 2:** Pre-test probabilities of obstructive coronary artery disease in 15 815 symptomatic patients according to age, sex, and the nature of symptoms in a pooled analysis of contemporary data

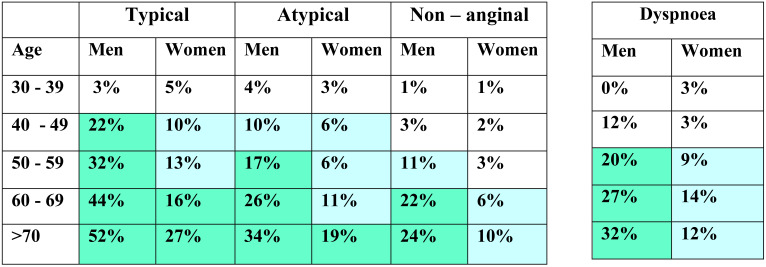

The regions shaded dark green denote the groups in which non-invasive testing is most beneficial (PTP >15%). The regions shaded light green denote the groups with PTPs of CAD between 5 and 15%, in which testing for diagnosis may be considered after assessing the overall clinical likelihood based on the modifiers of PTPs (Risk factors for CVD, abnormal resting ECG, left ventricular dysfunction, abnormal stress test and coronary calcium by CT)

The pivotal tests in patients presenting with clinical features of acute coronary syndrome are electrocardiography (ECG) and determination of serum troponin (cTn) I and/or T. Detection of a rise and/or fall of cTn with at least one value above the 99^th^ percentile upper reference limit and with at least one of the following: symptoms of acute myocardial ischemia, new ischemic ECG changes, development of pathologic Q waves, imaging evidence of new loss of viable myocardium or new regional wall motion abnormality in a pattern consistent with an ischemic etiology or identification of coronary thrombus by angiography, intracoronary imaging or autopsy (27).

**Optimal medical treatment**

The aim of this review is not to produce a detailed document on the medical management of CHD. However, a synopsis of the antiischemic, anti-thrombotic and other life saving measures should emphasize that a patient with ACS can only be safely managed in a setup equipped with facility for defibrillation; immediate measures like intravenous (IV) access, high flow oxygen, 12-lead ECG, continuous ECG monitor, IV analgesia, aspirin 300 mg and clopidogrel 600 mg; capacity to detect and manage complications like recurrent ischemia, heart failure and arrhythmia.

In CCS, optimal medical treatment includes non-pharmacologic therapy composed of heart-healthy diet, moderation of alcohol, cessation of smoking plus guideline directed treatment of hypertension & diabetes and anti-ischemic medications including beta blockers, calcium channel blockers, nitrates, ranolazine, trimetazidine and others. Until recently, cardiovascular event prevention had only been possible with aspirin and high intensity statin therapy.

Proprotein convertase subtilisin/kexin type 9 (PCSK9) is an enzyme which binds to LDL receptor triggering receptor degradation. PCSK9 inhibitors including alirocumab and evolocumab increase receptor density which in turn reduces LDL concentration. Serum LDL was reduced by more than 60% over and above high intensity statin treatment in studies that have proven safeties of these drugs, with outcome studies, a composite of cardiovascular death, non-fatal MI, stroke or unstable angina requiring admission was significantly reduced (28,29). This resulted in class I recommendation of these agents when the LDL target is not reached using a tolerated dose of a high intensity statin plus ezetimibe (30). The new dynamics of CVD prevention and treatment has also incorporated sodium-glucose co-transporter 2 (SGLT2) inhibitors to be part of the treatment. Empagliflozin, based on the results of the empagliflozin cardiovascular outcomes and mortality in Type 2 diabetes (EMPREG outcome) trial (31), is recommended to reduce risk of death in patients with diabetes and established CVD or those who are at high risk to develop it (26).

The outcome study results of canagliflozin and dapagliflozin (32, 33) demonstrated significantly reduced heart failure and other CVD endpoints. Both are, hence, recommended in patients with diabetes and CVD or high CVD risk to reduce cardiovascular events (34). Glucagon-like peptide 1 (GLP-1) agonists have been studied in outcome trials (35, 36, 37) showing liraglutide to reduce risk of death, while semaglutide and dulaglutide reduced risk of cardiovascular events. Likewise, the benefit of novel oral anticoagulants (NOACs) over and above aspirin has been studied (38, 39) with overall clinical benefit. In recent guidelines, rivaroxaban is a class IIa recommendation for patients at high risk of developing CVD events (26).

**Revascularization for ST-Segment Elevation Myocardial Infarction (STEMI)**

**Figure 4:**
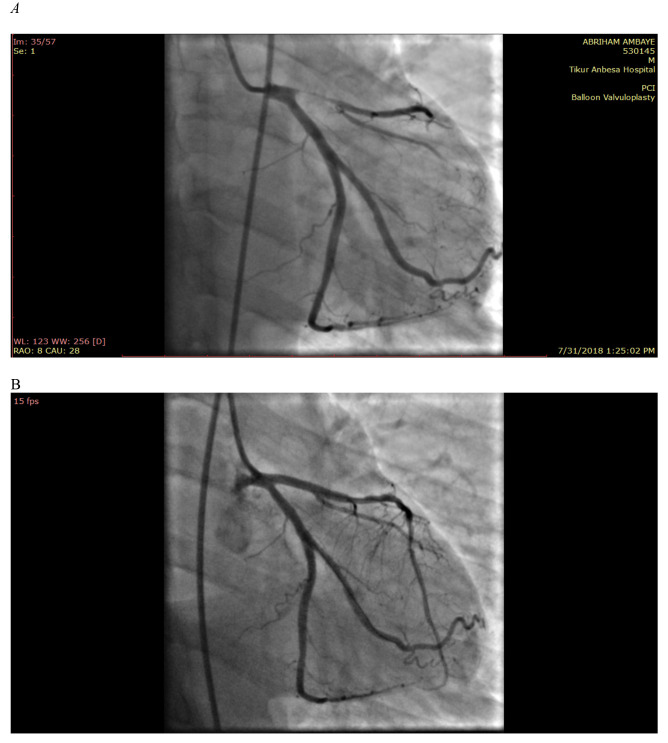
Left anterior descending coronary artery ostial occlusion resulting in STEMI: A. Before PCI, B. After PCI

Patients with ST-Segment Elevation Myocardial Infarction (STEMI) should receive reperfusion therapy either with primary PCI or fibrinolysis within 12 hours of the symptom onset to improve clinical outcome. A meta-analysis of initial studies of fibrinolysis demonstrated absolute mortality benefit of 3%, 2% and a non-significant 1% in patients presenting within 6 hours, 7–12 hours and 13–18 hours respectively (40). PCI is generally preferred for, patients with high risk of bleeding, high risk patients particularly those in shock, patients with diagnostic dilemma and failed fibrinolysis (rescue PCI). To determine the relative benefits PCI over fibrinolysis, several studies were done considering consecutive patients with STEMI, the meta-analysis of 23 such studies showing PCI to be superior to fibrinolysis with regards to all cause death, recurrent MI, total stroke, intracranial hemorrhage, and a composite of death, MI and stroke (41). Therefore, PCI is more effective, is associated with less recurrent ischemia, and it requires less hospital stay while it is more expensive and needs more experience (fig. 4). In those patients with failed fibrinolysis, rescue PCI is a viable strategy. The Rescue Angioplasty versus Conservative Treatment or Repeat Thrombolysis (REACT) trial divided 427 patients with failed primary fibrinolysis to rescue PCI or repeat administration of fibrinolysis. A composite outcome of death, recurrent MI, severe heart failure and stroke was significantly reduced in the PCI group at 6 months post procedure (42). A meta-analysis including 7 other studies concluded that recue PCI is superior to a conservative approach in terms of recurrent MI, heart failure, stroke and a composite of MI and stroke (43).

For effective revascularization, time since the onset of symptoms is critical. In the above stated meta-analysis comparing PCI with fibrinolysis (41) for every 10 minutes delay to PCI, there is 1% reduction in mortality difference versus fibrinolysis. Studies have been done aimed at determining the optimal door to balloon time (44,45) with a final conclusion that the advantages of PCI compared to fibrinolysis disappears as PCI-related delay increases to 114 minutes in settings with on-site PCI facility. However, even in the United States, PCI facility is limited to 25% of acute care hospitals (46) which prompted consideration of transfer of patients with STEMI to the nearest PCI center. The DANish trial in Acute Myocardial Infarction (DANAMI) trial demonstrated a significant, 40% reduction of a composite of death, recurrent MI and stroke, when patients were transferred for PCI than a repeat fibrinolytic treatment (47). The Trial of Routine Angioplasty and Stenting after Fibrinolysis to Enhance Reperfusion in Acute Myocardial Infarction (TRANSFER-AMI) study divided 1059 patients with STEMI between transfer to PCI center and conservative treatment with provision of rescue PCI. A primary outcome composed of death, recurrent MI and cardiogenic shock within 30 days was significantly lower in the group transferred for PCI (48). The maximum time delay still transfer of patients to the nearest PCI center remains beneficial was found to be 120 minutes in the National Registry of Myocardial Infarction (NRMI) (49). One study has also shown that biomarker elevation had the same extent and pattern between patients for whom PCI done within 3 hours of presentation and for those PCI was performed 3 to 12 hours after administration of tenecteplase (pharmacoinvasive strategy) with similar probability of death, non-fatal reinfarction, stroke or ischemia-driven revascularization (50).

With the intention of comparing pharmacoinvasive strategy with the results of primary PCI, in the Strategic Reperfusion Early after Myocardial Infarction (STREAM) trial, patients with STEMI who presented within 3 hours after symptom onset and who were unable to undergo primary PCI within one hour were randomly assigned to undergo either primary PCI or fibrinolytic therapy before transport to PCI-capable hospital. A composite primary end point of death, shock, congestive heart failure, or reinfarction up to 30 days occurred with the same frequency (51). Therefore, the ESC concluded that primary PCI is the preferred reperfusion strategy in patients with STEMI within 12 h of symptom onset, provided it can be performed within 120 min from STEMI diagnosis, otherwise patients should be transferred to a PCI-capable facility as soon as possible after a bolus of fibrinolytic for routine coronary angiography within 2–24 hours following fibrinolysis (52).

**Revascularization for Non-ST-Segment Elevation Myocardial Infarction (NSTEMI)**: In NSTEMI, given the pathophysiologic difference, the urgency and application of revascularization is different from STEMI. An invasive strategy has become a standard of care in high risk NSTEMI patients (53). Numerous factors interplay in the decision making process, including clinical presentation, comorbidities, risk stratification (54). Advantages of early intervention, include rapid and definitive evaluation, earlier revascularization may prevent further complications and facilitation of earlier discharge. Ischemia-guided strategy considers intervention with, failed medical treatment, objective evidence of ischemia and presence of high prognostic risk and to avoid costly, even possibly unnecessary, invasive procedures. Several studies have compared these two strategies and a meta-analysis of these studies revealed that a composite of cardiovascular death and MI was significantly low with routine invasive strategy. When patients were further stratified according to their risk profiles as, low, intermediate and high risk, the difference was restricted to high risk patients (55). Therefore, in resource limited countries, to which Ethiopia is not an exception, selection of patients for immediate invasive strategy should depend on risk assessment tools, like Thrombolysis In Myocardial Infarction (TIMI) or Global Registry of Acute Coronary Events (GRACE) risk scoring systems with emphasis on biomarker positivity.

**Revascularization in chronic coronary syndrome**: In CCS patients, the goals of treatment of CHD are 1) to reduce premature CVD death and to prevent complications 2) to restore a level of activity, functional capacity, and quality of life 3) to eliminate ischemic symptoms and 4) minimize costs of health care. The decision to proceed with revascularization of a certain vessel is reached with serious consideration of the extent of symptoms of the ischemic myocardium, the response to medical therapy, the likelihood of fatal or serious outcome in the event of abrupt vessel occlusion and suitability of the patient for coronary artery bypass grafting (CABG). Indications for coronary angiography with the intent of revascularization are the following. First, symptomatic improvement: persistence of symptoms despite medical treatment in that PCI or CABG is more effective in relieving angina, to reduce the use of anti-anginal drugs, to raise exercise capacity and quality of life. Second, improvement of prognosis: revascularization increases survival in a group of patients with CCS.

**Revascularization for chronic coronary syndrome to improve symptoms**: Starting early in its wide popularization, angioplasty was shown to be more effective in relieving angina compared to medical treatment. In the Angioplasty Compared to Medicine (ACME) trial percutaneous transluminal coronary angioplasty (PTCA) offered earlier and more complete relief of angina than medical treatment and it was associated with better performance on the exercise test (56). Contrary to this, recently, the Objective Randomised Blinded Investigation With Optimal Medical Therapy of Angioplasty in Stable Angina (ORBITA) trial randomly compared PCI with placebo (sham procedure) in patients with stable coronary artery disease (SCAD) due to single-vessel CAD (diameter stenosis >70%) and preserved left ventricular function in the presence of moderate symptoms of angina, Canadian Cardiovascular Society (CCS) class II in 59% of patients (57).

Following a 6-week post-randomization period, the primary endpoint of increment in exercise time was not significantly different between the two groups. ORBITA raises the issue of whether the symptom relief of PCI in the specific setting of stable single-vessel CAD may be related at least in part to a placebo effect. But, the dobutamine stress echocardiography peak stress wall motion score index improved with PCI compared to optimal medical treatment. Complete resolution of angina was also more frequent in PCI patients. Limitations of the study as acknowledged by the investigators include the short observation period (6 weeks), the inclusion of patients with mild symptoms (CCS class 0-I in 25% of patients), the group imbalance in ostial and proximal lesions (37 vs. 57%, P = 0.005), loss to follow-up patients after randomization, and the insufficient power to detect a true difference cannot be overemphasized (58). Besides, in the Fractional Flow Reserve-Guided PCI versus Medical Therapy in Stable Coronary Disease (FAME 2) trial, 888 patients with SCAD with reduced fractional flow reserve were randomly assigned to PCI plus medical treatment or medical treatment alone. The percentage of patients with CCS class II, III, or IV angina was significantly lower in the PCI group than in the medical treatment group at all-time points during 3 years of follow-up (59).

**Revascularization for chronic coronary syndrome to reduce CVD events or death**: In CCS, studies comparing a strategy of PCI with initial medical treatment found no or only modest benefits in terms of survival or MI for an invasive strategy. The Clinical Outcomes Utilizing Revascularization and Aggressive Drug Evaluation (COURAGE) trial was a randomized study involving 2287 patients who had objective evidence of myocardial ischemia and significant coronary artery disease. Between 1999 and 2004, 1149 patients were assigned to undergo PCI with optimal medical therapy and 1138 to receive optimal medical therapy alone. The primary outcome was death from any cause and nonfatal MI during a follow-up period of 2.5 to 7.0 years. Conclusion of the COURAGE trial was that as an initial management strategy, in patients with stable coronary artery disease, PCI did not reduce the risk of death, MI, or other major cardiovascular events when added to optimal medical therapy (60). A similar conclusion was drawn in the Bypass Angioplasty Revascularization Intervention 2 Diabetes (BARI 2D) trial in diabetic patients with SCAD (61).

However, none of the studies were adequately powered and crossover to revascularization occurred in high proportion (33% in the COURAGE trial and 42% in the BARI 2D trial) (62). Editorials of these studies commented that exclusion of high risk patients as evidenced by the inclusion of less than one third of patients in the COURAGE trial that had greater than 10% ischemia on myocardial perfusion study restrained firm and lasting conclusion (63). In these studies, the usage of drug eluting stents was limited to less than 40 %, whereas, revascularization techniques have matured over time to the era of third generation stents resulting in clinical outcome benefits.

To determine the effect of adding coronary angiography and revascularization based on contemporary practice pattern International Study of Comparative Health effectiveness with Medical and Invasive Approaches (ISCHEMIA) trial was designed. The ISCHEMIA trial randomly assigned 5179 patients with stable CAD, in a 1:1 ratio to an initial invasive strategy of medical therapy, angiography, and revascularization considered when feasible or to an initial conservative strategy of medical therapy alone, with angiography reserved for failure of medical therapy. Included patients age was above 20 with moderate to severe ischemia as evidenced by: myocardial perfusion study ≥ 10 % left ventricular ischemia, stress echocardiography ≥ 3 segments showing moderate or severe hypokinesis, or akinesis, with Cardiac Magnetic Resonance imaging (CMR) ≥ 12% ischemia, Exercise Tolerance Testing (ETT) ≥1.5 mm ST-segment depression in 2 leads or ≥ 2 mm ST-segment depression in a single lead at ≤ 7 Metabolic Equivalent (MET). The primary outcome was a composite of death from cardiovascular causes, MI, or hospitalization for unstable angina, heart failure or resuscitated cardiac arrest. After a median follow up 3.2 years, the investigators concluded that among patients with SCAD and moderate or severe ischemia, they did not find evidence that an initial invasive strategy, as compared with an initial conservative strategy, reduced the risk of ischemic cardiovascular events or death from any cause. Procedural MI was high in the invasive group while spontaneous MI was significantly higher in the initial medical therapy group (64).

Nevertheless, previous studies had shown that, performing PCI on non-ischemic stenosis is not beneficial (65), and is probably harmful (66). Thus, careful selection of ischemia-inducing stenosis is essential for deriving the greatest benefit from revascularization in patients with SCAD. The Fractional Flow Reserve versus Angiography for Multivessel Evaluation (FAME) study divided 1005 patients with multi-vessel coronary artery disease to undergo PCI with implantation of drug-eluting stents guided by angiography alone or guided by Fractional Flow Reserve (FFR) measurement in addition to angiography. The rate of the composite end point of death, nonfatal MI, and repeat revascularization at 1 year was significantly reduced with routine measurement of FFR (67).

This initiated designing a study with aim of determining whether FFR-guided PCI with drug-eluting stents plus the best available medical therapy is superior to the best available medical therapy alone in reducing the rate of death, MI, or unplanned hospitalization leading to urgent revascularization among patients with CCS. The Fractional Flow Reserve versus Angiography for Multivessel Evaluation 2 (FAME 2) study halted recruitment prematurely because FFR-guided PCI plus the best available medical therapy, as compared with the best available medical therapy alone, decreased the need for urgent revascularization (59). A network meta-analysis of 100 trials with 93553 patients and 262090 patient-years of follow-up comparing a strategy of initial medical therapy with revascularization reported improved survival using PCI with new generation DES compared with initial best available medical treatment (68).

Commentaries made on the ISCHEMIA trial emphasized on the following issues, 1) it was already known from randomized trials like the COURAGE trial that revascularization does not swing the pendulum of mortality in CCS, 2) the rigorous inclusion criteria of ISCHEMIA, trial patients underwent both anatomical testing (CCTA) and functional testing (nuclear imaging), unusual in routine clinical practice, 3) potential effect of the practice pattern may have excluded more symptomatic patients from the trial in countries with a low threshold for revascularization, 4) the composite outcome was initially designed to include CVD death and MI, to have more events, unstable angina, heart failure and resuscitated cardiac arrest were included, 5) there was divergence of the curve favoring the invasive strategy at 4 years (69). Moreover, limitations of the ISCHEMIA trial as acknowledged by the investigators were, 1) power of the study was decreased by reducing the sample size from 8000 to 5179, 2) event rate was lower than expected, 3) the period of follow-up was modest, long-term follow-up with assessment of mortality is needed to fully understand the prognostic implications of more procedural and fewer nonprocedural infarctions with an invasive strategy.

The implications all of these need serious consideration before fully absorbing the findings of the ICHEMIA trial to the real world practice. Therefore, it is difficult to draw a definite conclusion different from the existing standard recommendation of revascularization with the purpose of improving clinical outcome in: 1) left main stenosis, 2) three-vessel CAD, particularly with ejection fraction of less than 40%, 3) two vessel disease with more than 75% stenosis of the proximal LAD. The role of revascularization to eliminate symptoms and to reduce physical limitation remains also decisive.

**The status of coronary revascularization in Ethiopia and the way forward**: Coronary revascularization is now a recognized standard procedure among the medical community in Ethiopia with PCI being performed in several places in Addis Ababa and in one public hospital in Mekelle while CABG is just starting to popularize. The journey of coronary revascularization has been embraced by young health professionals within the country and experts from abroad who have helped to start the procedures seamlessly. This dragged the interests of some institutions and organizations that have significantly impacted on the development of coronary intervention. All those contributions can be categorized into four distinct players.

First, the performance of the first coronary angiography and PCI at Addis Cardiac Hospital in 2007 by the author of this article, Dr Bekele Alemayehu Shashu, after the establishment of the first catheterization laboratory (cath-lab) in the country by the Ethiopian born Swedish cardiologist, Dr FikruMaruWordofa, was a milestone achievement. This inspired young physicians drawing their devotion to cardiology against the scepticism of senior colleagues. Second, the subsequent establishment of Cardiac Centre Ethiopia by Children's Heart Fund of Ethiopia, a local nongovernmental organization, through the unremitting work of Dr Belay AbegazMolla played a pivotal role in further promoting the procedures. The center had been organizing surgical and interventional missions from outside until the training of the outstanding cardiac surgens and interventional cardiologists abroad was accomplished that converted the charity based missions into standing programs entirely covered by the local staff. Third, cardiology and cardiothoracic surgery fellowship programs at the College of Heath Science, Addis Ababa University, in TikurAnbessa Specialized Hospital and the cardiology fellowship program at the Millennium Medical School, in St Paul's Hospital, further broadened the effort providing it an academic contour. Fourth, the Society of Cardiac Professionals of Ethiopia under the leadership of the first president, Dr Dejuma Yadeta Goshu, has been active to offer a platform of continuing medical education (CME) engaging experts in the field locally and from abroad which significantly contributed to implementing guideline directed treatment in cardiology. This together with the enthusiastic academic practices at the medical schools resulted in an unofficially concerted effort among the cardiac professionals in the country. It is; therefore, believed that concrete steps have been achieved in the field of cardiology in general and in coronary revascularization in particular in Ethiopia while the challenge ahead is tremendous. The government under the proactive role of the Ministry of Health of the Federal Democratic Republic of Ethiopia is to acknowledge the efforts underway in cardiology and other state-of-the-art medical practices. This would enable participatory and inclusive insurance system to cover the reimbursement of the cost of consumables and devices. Acute MI especially extensive STEM claims the lives of middle aged professionals, politicians and the business community who are important players in the country's economy. The foreign currency loss for such expensive procedures abroad is so distressing that the country can't afford to continue with. Pharmacoinvasive strategy through careful selection of cases at each level in the tier of clinical care is the way forward which should start through supplying fibrinolytic drugs, at least streptokinase, in hospitals with internists across the Ethiopia. This reinforced by organized primary preventive practices would enable the country to decrease the multifaceted loss to which it's immensely exposed.

In conclusion, coronary revascularization is a standard procedure saving millions of lives every year globally. Timely presenting patients with STEMI should undergo primary PCI within 120 minutes. Those who are unable to have PCI within the time frame provided require fibrinolytic drugs with clear plan of pharmacoinvasive strategy in place. In NSTEMI acute coronary syndromes, selection of patients for immediate invasive strategy should depend on risk assessment tools. The indications for intervention in CCS are 1) left main stenosis, 2) three-vessel CAD, particularly with ejection fraction of less than 40%, 3) two vessel disease with more than 75% stenosis of the proximal LAD. It's also applicable to eliminate symptoms and to reduce physical limitations. Coronary revascularization is considerably popular procedure in Ethiopia while its sustainability relies on the government's attention to minimize the multifaceted problem.
